# Differential activation of RAW 264.7 macrophages by size-segregated crystalline silica

**DOI:** 10.1186/s12995-016-0145-2

**Published:** 2016-12-15

**Authors:** Steven E. Mischler, Emanuele G. Cauda, Michelangelo Di Giuseppe, Linda J. McWilliams, Claudette St. Croix, Ming Sun, Jonathan Franks, Luis A. Ortiz

**Affiliations:** 1 National Institute for Occupational Safety and Health, Office of Mine Safety and Health Research, 626 Cochrans Mill Road, Pittsburgh, PA 15236 USA; 2 Department of Environmental and Occupational Health, University of Pittsburgh, Pittsburgh, PA USA; 3 Center for Biological Imaging, Environmental and Occupational Health, University of Pittsburgh, Pittsburgh, PA USA; 4 Center for Biological Imaging, University of Pittsburgh, Pittsburgh, PA USA

**Keywords:** Ultrafine crystalline silica, Alveolar macrophage activation, Size segregation, Occupational aerosols

## Abstract

**Background:**

Occupational exposure to crystalline silica is a well-established occupational hazard. Once in the lung, crystalline silica particles can result in the activation of alveolar macrophages (AM), potentially leading to silicosis, a fibrotic lung disease. Because the activation of alveolar macrophages is the beginning step in a complicated inflammatory cascade, it is necessary to define the particle characteristics resulting in this activation. The aim of this research was to determine the effect of the size of crystalline silica particles on the activation of macrophages.

**Methods:**

RAW 264.7 macrophages were exposed to four different sizes of crystalline silica and their activation was measured using electron microscopy, reactive oxygen species (ROS) generation by mitochondria, and cytokine expression.

**Results:**

These data identified differences in particle uptake and formation of subcellular organelles based on particle size. In addition, these data show that the smallest particles, with a geometric mean of 0.3 μm, significantly increase the generation of mitochondrial ROS and the expression of cytokines when compared to larger crystalline silica particles, with a geometric mean of 4.1 μm.

**Conclusion:**

In summary, this study presents novel data showing that crystalline silica particles with a geometric mean of 0.3 μm enhance the activation of AM when compared to larger silica particles usually represented in in vitro and in vivo research.

## Background

Occupational exposure to crystalline silica (CS) affects at least 1.7 million US workers [1] and is associated with the development of silicosis, a fibrotic lung disease which is one of the most important occupational diseases worldwide [2–4]. The National Institute for Occupational Safety and Health (NIOSH) reported that 300 silicosis-related deaths occurred each year in the United States between 1991 and 1995 [5]. During those same years China recorded 24,000 silicosis-related deaths per year [6]. These numbers indicate that silicosis remains a fundamental occupational exposure problem in both the developing and developed countries [7].

Exposure to CS occurs in many occupations and industries. The United States Occupational Safety and Health Administration (OSHA) measured detectable levels of respirable CS in samples collected in 255 different industries [1]. In general, silica exposure will occur in any occupation that includes grinding or mechanically breaking material containing silica (mining, construction) or handling fine particles containing silica, such as silica sand (fracking) [4, 8–12].

Although occupational exposure to CS and the related health effects have been well documented in the scientific literature, many uncertainties still exist including the effect of the crystals’ surface characteristics, including particle size, on the development of disease [8, 13–18]. Most atmospheric studies suggest that the concentration of smaller particles correlates better with adverse health effects than the concentration of larger particles [16, 19–22]; however, there is little size-dependent toxicity data concerning CS. One difficulty in completing size-dependent toxicity studies with CS is the difficulty in separating the occupational aerosol into distinct size ranges and in necessary quantities for toxicological studies. Recently our group published research on a novel multi-cyclone sampling array which enables the separation of occupational aerosols into distinct size ranges and in quantities needed for toxicological research [23].

Because smaller particles have a higher surface area per unit mass when compared to larger particles, smaller particles may more readily initiate potential negative biological reactions, such as inflammation [24]. Chronic inflammation has been implicated in the pathogenesis of silicosis. In this scenario, the immune cells (alveolar macrophages, epithelial cells, and fibroblasts) are activated and release a host of inflammatory cytokines and generate reactive oxygen species (ROS), resulting in the recruitment of additional inflammatory cells, predominantly alveolar macrophages. The influx of additional inflammatory cells and release of ROS damages pulmonary architecture, causing accumulation of connective tissue products [7, 14, 25–27]. Knowledge of the degree to which particle size affects the activation of macrophages and the resulting ROS generation and inflammatory response is necessary for fully elucidating the mechanisms leading to silicosis from occupational exposure to crystalline silica.

In the present in vitro study, the macrophage response to different-sized crystalline silica particles was evaluated in a well-established murine model [28, 29] using the mouse monocyte-macrophage RAW 264.7 cell line. Airborne CS particles were separated into four distinct size ranges using the multi-cyclone sampling array. The RAW 264.7 macrophages (AM) were exposed to four different sizes of CS and their activation was measured using electron microscopy, mitochondrial ROS (mROS) generation, and cytokine expression.

## Methods

### Particles used for method evaluation

The crystalline silica (SiO_2_ quartz, 99.9%, 1 μm, Stock #: 4807YL) used in this study was purchased from Nanostructured & Amorphous Materials, Inc. (Houston, TX). Before particles were used in the experiment they were baked at 220 °C for 24 h to destroy potential contaminating endotoxins.

### Particle separation

The CS particles used in this study were separated into four distinct size ranges as presented in Table 1– ultrafine (UF), submicron (S), respirable (R), and coarse (C)–using a multi-cyclone sampling array (MCSA).

**Table 1:** Mean particle diameter for each size range using DLS/LLS data

Particle name	Mean (μm)	Standard deviation
Coarse (C)	4.092	2.386
Respirable (R)	2.123	1.146
Submicron (S)	0.716	0.152
Ultrafine (UF)	0.294	0.098

**Fig. 1:**
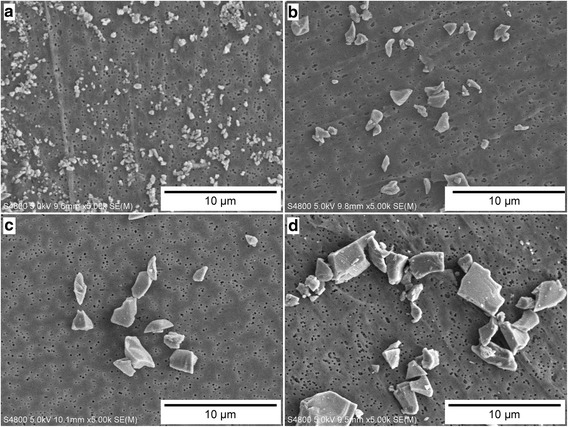
Comparison of SEM images of the four sizes of crystalline silica particles used for this study, (**a**) Ultrafine (UF), (**b**) Submicron (S), (**c**) Respirable (R), and (**d**) Coarse (C). Images are all at the same magnification (5,000x)

The MCSA was described in detail previously [23] and particle size ranges are named for consistency with that publication and do not match other established naming conventions found in the literature. Briefly, the MCSA incorporates cyclones in a series of three successive stages. Each cyclone stage is used to capture a specific size range of particles, in decreasing sizes with each stage in the series. The innovative idea in this design is to use the particles collected in the cone and grit pot of each cyclone for characterization and analysis. In this method, three cyclone stages and one filter stage (after the final cyclone) were used to collect size-segregated CS particles. The exhaust from one cyclone was connected to the inlet of the next cyclone in the series using conductive tubing. The mean particle diameter and standard deviation for each size range, measured using light scattering techniques (DLS/LLS), is presented in Table 1 and scanning electron microscope (SEM) photographs at 5,000x magnification of each size range are presented in Fig. 1.

### Particle extraction from MCSA

The size-segregated particles were collected from the grit pot of each cyclone using the procedure described in detail in Mischler et al. [23]. Briefly, the grit pot was first inverted and tapped on the outside, allowing the particles to fall out of the grit pot and be collected onto an aluminum collection disk. Any particles which did not fall from the grit pot or particles which remained in the cone were scraped from these surfaces using a plastic or wooden scraping tool. Once the particles were recovered from each cyclone stage, they were stored for later characterization and testing.

The pre-weighed filter, containing the smallest size range of particles, was post-weighed to determine the mass of particles collected on the filter. The particles collected on the filter were then removed using the following procedure: 1) The filter containing the particles was placed in a 15-ml centrifugal test tube, submerged in 5 ml of ultrapure deionized water, and sonicated for five minutes; 2) after sonication, the filter was removed from the first tube and placed in a second tube. Five ml of ultrapure deionized water was added to the second tube, and the filter was submerged in the water and sonicated for five minutes; 3) after sonication, the water was poured into the first tube, which now contained 10 ml of particle-laden water; 4) another 5 ml of ultrapure deionized water was then added to the tube containing the filter, and the filter was submerged into the water and sonicated for five minutes; 5) after sonication, the 5 ml of water was poured into the first tube, which now contained 15 ml of particle-laden water; 6) the tube containing the 15 ml of particle-laden water was then centrifuged for seven minutes at 3901 relative centrifugal force (RCF); 7) after centrifugation, the supernatant was removed leaving the particles at the bottom of the tube; 8) serum-free Dulbecco’s Modified Eagle’s Medium (DMEM) was then added to the tube containing the particles to achieve the desired exposure concentration, with the necessary volume for the addition of DMEM calculated based on the post-weight of the filter; 9) once the particles were removed from the filter, the filter was dried and reweighed to verify the mass of particles removed. During this study, the average removal efficiency of the ultrafine CS particles from the filter was 90% as reported previously [23].

### Silica particle suspension and characterization

Stock silica suspensions were prepared by adding serum-free DMEM into a measured mass of crystalline silica particles to achieve the desired particle concentrations. For cytokine analysis an exposure concentration of 50 μg/cm^2^ was used for all particle sizes, for live-cell experiments and transmission electron microscopy (TEM) analysis an exposure concentration of 16.6 μg/cm^2^ was used for all particle sizes. The difference in silica concentrations between these two analyses was necessary because the 50 μg/cm^2^ CS concentration completely covered the cells, preventing collection of TEM and live-cell data. 16.6 μg/cm^2^ was the highest concentration of UF CS which did not prevent TEM and live-cell data collection. The silica suspensions were used within two hours of mixing with serum-free DMEM. Before each exposure the stock solutions were sonicated for 15 min to ensure adequate distribution of the particles and reduce any agglomeration. After exposure the stock solutions were analyzed with dynamic light scattering, a broadly used and widely accepted method for measuring particle size in solution, to verify particle size distribution, as described previously [23].

### Cell cultures

Cells from the mouse monocyte-macrophage RAW 264.7 cell line were purchased from American Type Tissue Culture Collection (ATCC, Rockville, MD) and maintained according to ATCC protocols at 37 °C in a 5% CO_2_/95% air humidified incubator. Cells were cultured in DMEM with 10% fetal bovine serum (FBS) and penicillin-streptomycin, on 75-cm^2^ plates. Approximately 24 h prior to exposure the cells were plated into 6- or 12-well plates. Cells were seeded at a concentration of 1.05 × 10^5^ cells/cm^2^ in the indicated culture dishes and the exposure experiments were completed when the cells were at 80% confluency. The use of RAW 264.7 cells is an established murine model in the literature for crystalline silica experiments [28, 29].

### TEM analysis

At one, two, and four hours after exposure, cells were fixed in 2.5% glutaraldehyde in phosphate-buffered saline (PBS) and post-fixed in 1% osmium tetroxide in PBS, dehydrated through a graded series of alcohols and embedded in Epon (Energy Beam Sciences, Agawam, MA). Thin (70-nm) sections were cut using a Reichert Ultracut S (Leica, Deerborn, MI), mounted on 200-mesh copper grids and counter-stained with 2% aqueous uranyl acetate for seven minutes and 1% aqueous lead citrate for two minutes. Observation was with a JEOL 1011 transmission electron microscope (Peabody, MA). After TEM images were collected, they were formatted using Adobe Photoshop for brightness and contrast. In addition, during slide preparation, a CS particle could create stretching in the epon resin during the slicing sequence, and when stretching was severe the CS particle could fall out of the resin. During TEM imaging, any areas where the CS particles fell out will show as bright white, causing difficulty in image focusing. These images were corrected by re-coloring the white areas back to the color of the silica particles. Importantly, this may result in a slight increase in the CS particle size for the areas that were recolored. In the images at higher magnification, these areas are labelled.

### Live-cell analysis

Cells were seeded on 35-mm glass bottom dishes (MatTek Corporation, Ashland, MA) and incubated with the superoxide indicator MitoSOX™ Red (5 μM, Invitrogen, Eugene, OR) for 15 min at 37 °C. Cells were washed with PBS, the media was replaced with exposure media, and the dish was inserted into a closed, thermo-controlled (37 °C) stage top incubator (Tokai Hit Co., Shizuoka-ken, Japan) atop the motorized stage of an inverted Nikon TiE fluorescent microscope (Nikon Inc., Melville, NY) equipped with a 60X oil immersion optic (Nikon, CFI PlanFluor, NA 1.43) and NIS Elements Software. MitoSOX™ Red was excited using a Lumencor diode-pumped light engine (SpectraX, Lumencor Inc., Beaverton OR) and detected using a DsRed longpass filter set (Chroma Technology Corp) and ORCA-Flash4.0 sCMOS camera (HAMAMATSU Corporation, Bridgewater, NJ). Data was collected on approximately 80 to 100 cells per stage position, with eight to ten stage positions in each of the separate experiments for 180 min. Data were analyzed using NIS Elements (Nikon Inc., Melville, NY). Data from three independent analyses for each particle size were used in the statistical calculations. Stage positions in which the particles did not result in alveolar macrophage (AM) generation of ROS were not used in the statistical analysis.

### Cytokine analysis

The effect of particle size on expression of inflammatory cytokines was evaluated using the Bio-Plex multiplex magnetic bead technology (LMC0001, Mouse Cytokine 20-Plex, Invitrogen/Life Technologies, Carlsbad, CA). This assay simultaneously measured the concentration of 20 cytokines in the cellular supernatant. At least three independent experiments were conducted for cytokine expression. Each experiment was conducted using a nested triplicate model where each exposure was run in triplicate and each sample was analyzed in triplicate. For every experiment both a positive and negative control were used. For the negative controls the cells were treated only with serum-free DMEM medium. For the positive control, the cells were treated with serum-free DMEM containing 200 ng/ml of LPS. The effect of CS particles on murine macrophages using control particles such as titanium dioxide or carbon black, is well described in the literature and thus was not repeated in these experiments [30]. In each experiment, cells were exposed to one of four different CS particle stock solutions, described above, for two, four, or eight hours prior to collection of the supernatant. After collection, the supernatant was stored at −80 °C until analysis.

### Statistical analysis of ROS measurements

To help understand how CS particle size affects the production of reactive oxygen species in the mitochondria (mROS) in AM, AM were exposed to both UF and C particles over a 3-h exposure period and mROS production was measured using the superoxide indicator MitoSOX™ Red.

In this study, fluorescence was measured at approximately three-min intervals over a period of 181 min in samples exposed to UF and C particles. Measurements were taken on three samples exposed to each of the two particle sizes at each of the 61 time points. For the purpose of data analysis, the 61 time points were grouped into 30-min periods as shown in Table 2, resulting in a larger sample size for each statistical test, and therefore greater statistical power. Data were analyzed by six independent-samples t-tests comparing mean fluorescence values across the two conditions for each time period. A *p*-value less than 0.05 was considered to be statistically significant.

**Table 2:** Grouping of time points for ROS analysis

Time Period	Time points (minutes)	Number of Time Points	Number of Data Points per Condition
1	1, 4, 7, 10, 13, 16, 19, 22, 25, 28, 31	11	33
2	34, 37, 40, 43, 46, 49, 52, 55, 58, 61	10	30
3	64, 67, 70, 73, 76, 79, 82, 85, 88, 91	10	30
4	94, 97, 100, 103, 106, 109, 112, 115, 118, 121	10	30
5	124, 127, 130, 133, 136, 139, 142, 145, 148, 151	10	30
6	154, 157, 160, 163, 166, 169, 172, 175, 178, 181	10	30

Because examination of distributions of fluorescence, using the Shapiro-Wilk test, showed that the values did not follow a normal distribution, data were also analyzed using the Wilcoxin test, which is the non-parametric counterpart of the *t*-test. However, since results of the Wilcoxin test agreed with the results of the *t*-test in every case, only results of the t-tests are reported.

### Statistical analysis for cytokine expression

Nonparametric statistical tests, which make no assumption about the distribution of the data, were used to investigate differences of cytokine expression from exposure to the different particle sizes. A *p*-value less than or equal to 0.10 was considered to be statistically significant to ensure all potentially significant differences were identified. *P*-values of greater significance are noted in the figures and tables. The Kruskal-Wallis test is the nonparametric alternative to the one-way analysis of variance (ANOVA). This test was used to compare mean ranks among all the samples. When the results of this test were statistically significant the null hypothesis of no difference was rejected and post-hoc tests were run to search for pair-wise differences. For the latter analysis, the Wilcoxon rank sum test, the nonparametric analogue to the *t*-test for independent samples was used. For both the Kruskal-Wallis and Wilcoxon tests, exact probabilities were calculated [31].

## Results

### TEM Images of RAW cells after exposure with two sizes of crystalline silica

In order to visualize the difference in the handling of UF particles and C particles by AM, RAW 264.7 AM were exposed to UF and C particles for one, two, and four hours. At the appointed time the cells were fixed and stored until image collection, as discussed in the Methods section.

**Fig. 2:**
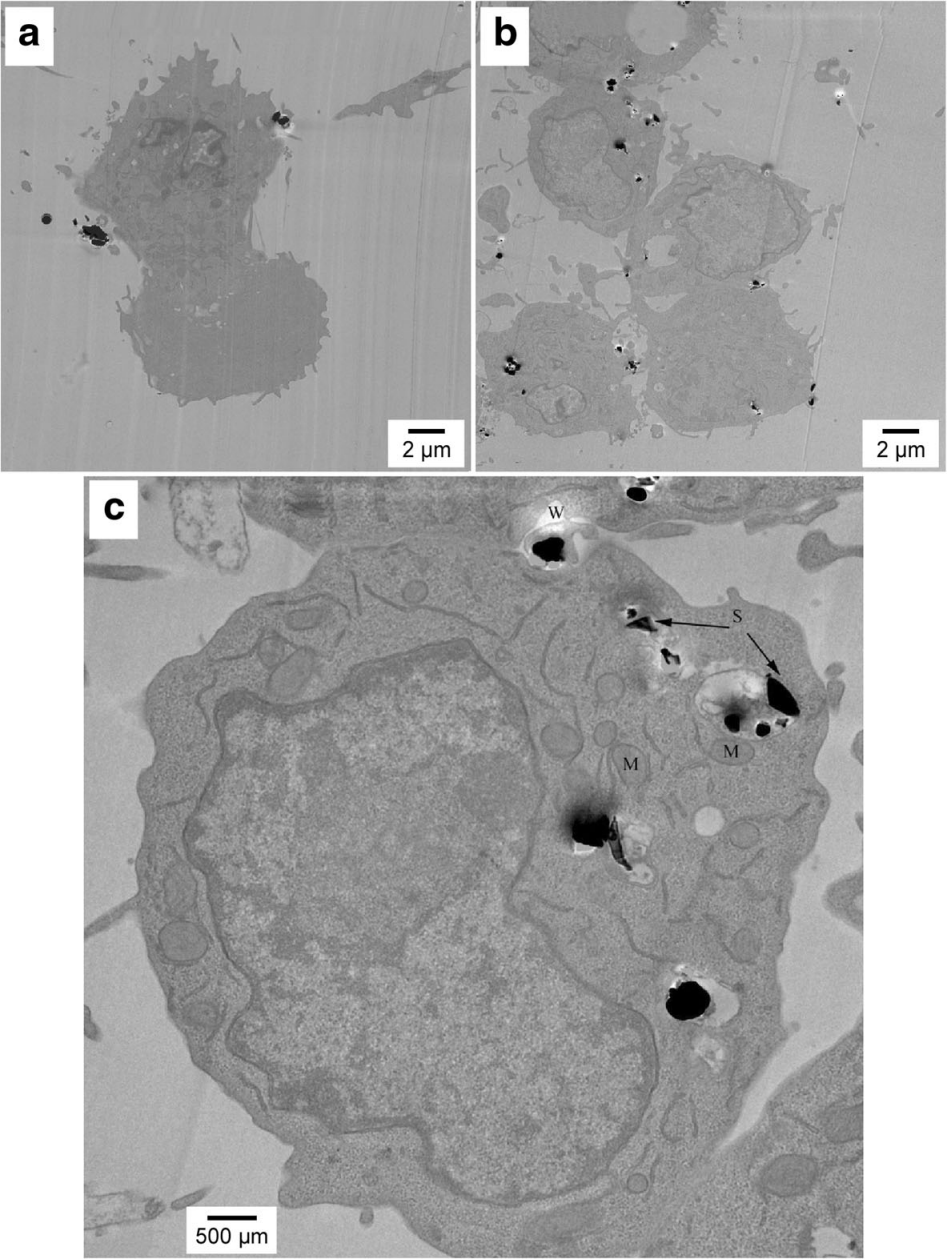
TEM images of AM after 1-h exposure of AM to (**a**) Coarse particles at 6,000x magnification, (**b**) UF particles at 6,000x magnification, and (**c**) UF particles at 20,000x magnification. In the higher magnification micrograph, the silica particles are marked S, the mitochondria are marked M, and W denotes a white area resulting from particles falling out of the resin. Figures present a representative image of the differences in AM uptake and handling of different sized crystalline silica particles

Figure 2a and b present images (6,000x magnification) of the AM cells after exposure for one hour to C and UF particles, respectively. At this time point, the images show that the UF particles are internalized more quickly and in larger numbers than the coarse particles. This result is consistent with the literature showing faster uptake of UF particles by AM. In addition, Figure 2b shows phagolysosome (PL) swelling is starting to occur in response to the UF particles. Figure 2c is a higher magnification (20,000x) image of an AM exposed to UF particles for one hour. In this micrograph, the silica particles are marked S, the mitochondria are marked M, and W denotes a white area resulting from particles falling out of the resin, as discussed in the Methods section. In Fig. 2c there is no evidence of UF particles in the cytoplasm or mitochondria.

**Fig. 3:**
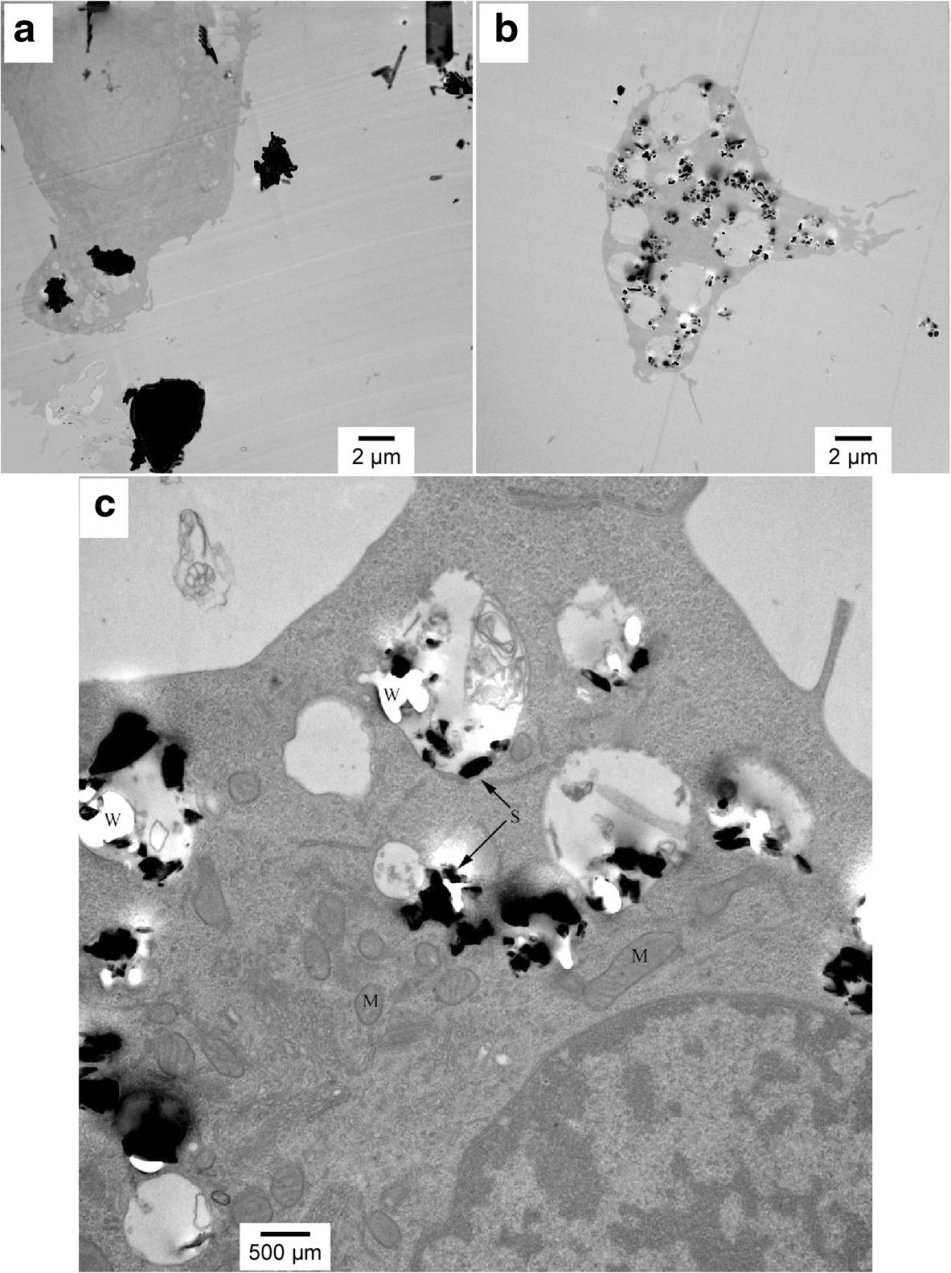
TEM images of AM after 2-h exposure of AM to (**a**) Coarse particles at 6,000x magnification, (**b**) UF particles at 6,000x magnification, and (**c**) UF particles at 20,000x magnification. In the higher magnification micrograph, the silica particles are marked S, the mitochondria are marked M, and W denotes a white area resulting from particles falling out of the resin. Figures present a representative image of the differences in AM uptake and handling of different sized crystalline silica particles

Figure 3a and b present images (6,000x magnification) of the AM cells after exposure for two hours to C and UF particles, respectively. These micrographs also show that a larger number of UF particles have been internalized by this time point than the C particles, once again consistent with the literature. Figure 3a shows that by the two-hour time period the C particles have been phagocytized by the AM and PL swelling is apparent. Figure 3b shows a cell with up to ten large PLs each containing numerous UF particles. In this figure the PLs are clearly swollen, indicating acidification and enzyme and protease production and delivery. For the C particles, each AM is seen to have phagocytized between one and three particles, whereas as many as 100 UF particles have been phagocytized by this time point. Figure 3c is a higher magnification image of an AM exposed to UF particles for two hours and shows that each PL contains numerous particles. In Fig. 3c there is no evidence of UF particles in the cytoplasm or mitochondria.

**Fig. 4:**
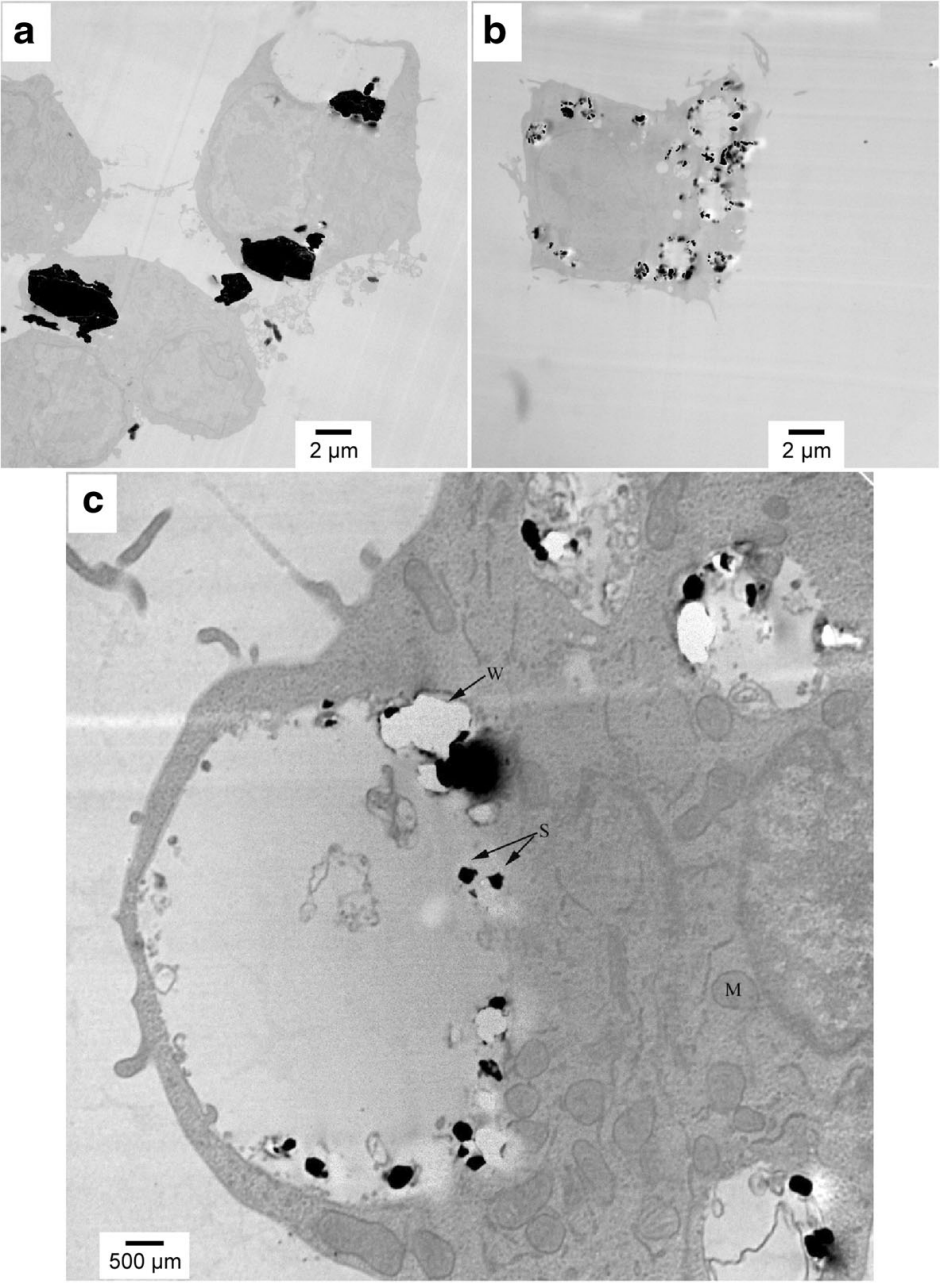
TEM images of AM after 4-h exposure of AM to (**a**) Coarse particles at 6,000x magnification, (**b**) UF particles at 6,000x magnification, and (**c**) UF particles at 20,000x magnification. In the higher magnification micrograph, the silica particles are marked S, the mitochondria are marked M, and W denotes a white area resulting from particles falling out of the resin. Figures present a representative image of the differences in AM uptake and handling of different sized crystalline silica particles

Figure 4a and b present images (6,000x magnification) of the AM cells after exposure for four hours to C and UF particles, respectively. These images are similar to two-hour images, where the UF particles have been phagocytized in greater number, resulting in creation of a higher number of PLs. The PLs are swollen but the membrane appears to still be intact. This can be seen more clearly in Fig. 4c, a higher magnification image of an AM exposed to UF particles for four hours. In Fig. 4c there is no evidence of UF particles in the cytoplasm or mitochondria.

### Generation of mitochondrial ROS

**Fig. 5:**
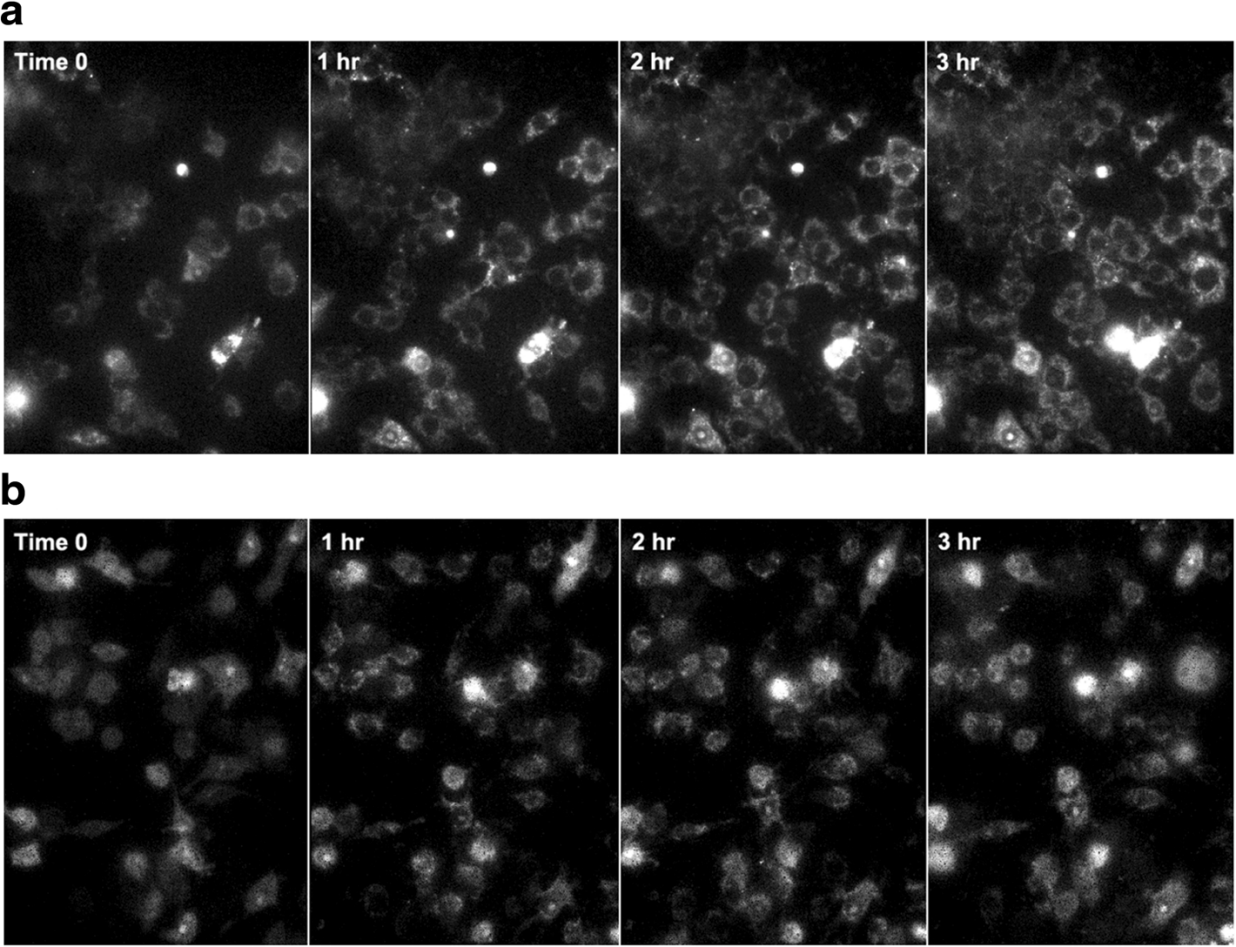
To help understand how particle size affects the production of reactive oxygen species in the mitochondria (mROS) in AM we exposed AM to both UF (**a**) and C (**b**) particles over a 3-h exposure period and measured mROS production using the superoxide indicator MitoSOX™ Red. mROS production is demonstrated over three hours in these representative images using original magnification x10, of the live cell experiment collected at 0, 1, 2 and 3 h, by increase in fluorescence

**Fig. 6:**
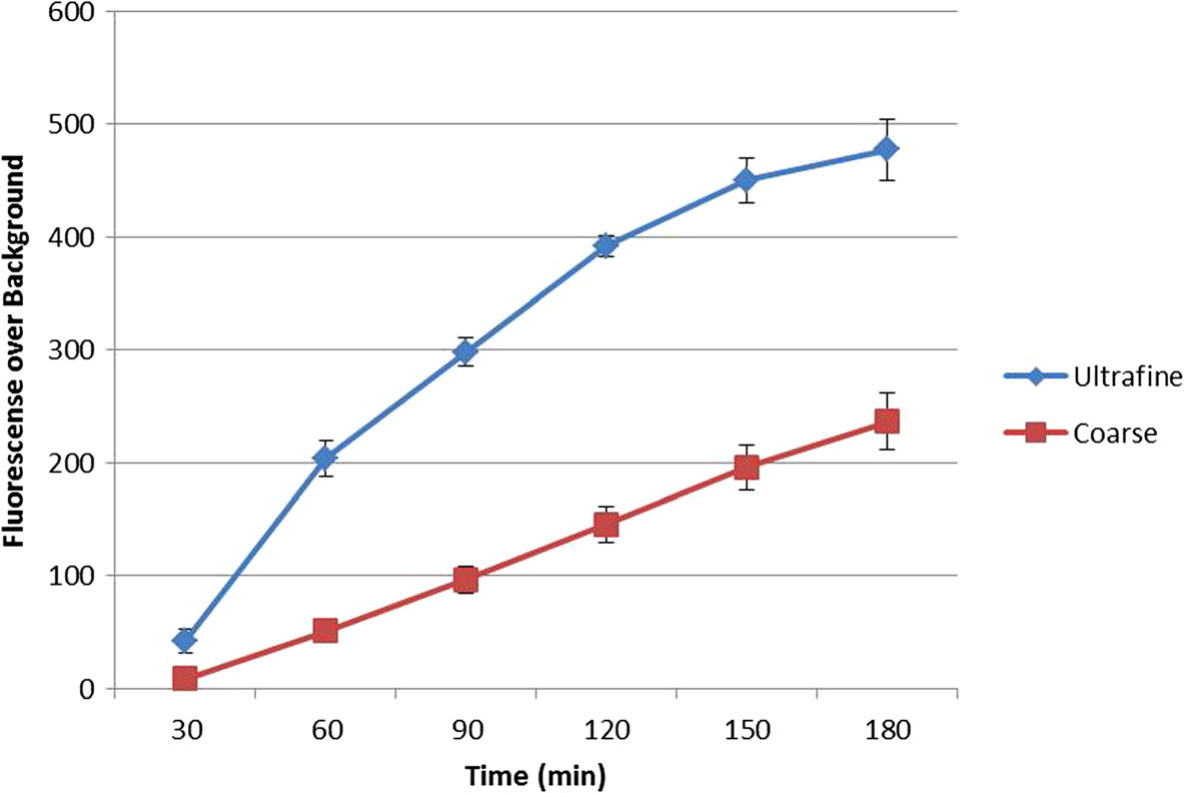
Increase in AM mROS production resulting from exposure to UF particles, when compared to coarse particles, was confirmed using MitoSOX™ Red fluorescence. Data was collected on approximately 80 to 100 cells per stage position, with eight to ten stage positions in each of the separate experiments for 180 min. Data were analyzed using NIS Elements (Nikon Inc., Melville, NY). Data from three independent analyses for each particle size were used in the statistical calculations. Stage positions in which the particles did not result in AM generation of ROS were not used in the statistical analysis

To help understand how CS particle size affects AM activation, the production of mROS in AM in response to exposure to C and UF particles was measured over a three-hour period. Means and standard deviations of MitoSOX™ Red fluorescence measurements, t-values, and *p*-values are shown in Table 3. The assumption of equality of variances was not met for several of the tests, and in those cases *p*-values associated with the Satterthwaite approximation of degrees of freedom are reported. Figure 5 is a representative image from the live-cell experiments taken at 0, 1, 2, and 3 h, for both UF (5A) and C (5B) particles. Increase in mROS can be seen as an increase in fluorescence. Figure 6 is a graph of the means reported in Table 3, and shows the increase in AM mROS production resulting from exposure to UF particles, when compared to coarse particles.

**Table 3:** Results of independent samples t-tests for mROS production using MitoSOX™ Red fluorescence measurements

Time Period	Particle Size	Mean	SD	t	*p*
1	Ultrafine	42.55	60.84	3.12	.004^*^
	Coarse	8.98	11.35		
2	Ultrafine	203.81	87.86	8.76	<.0005^*^
	Coarse	51.02	32.47		
3	Ultrafine	297.86	67.92	11.54	<.0005
	Coarse	96.64	67.19		
4	Ultrafine	391.79	49.32	13.43	<.0005^*^
	Coarse	145.69	87.42		
5	Ultrafine	450.25	108.07	9.15	<.0005
	Coarse	196.02	107.24		
6	Ultrafine	477.14	151.41	6.41	<.0005
	Coarse	236.56	139.18		

^*^ Based on Satterthwaite approximation

As can be seen in Table 3, differences between the UF and C particle size conditions were significant at the 0.01 level for Time Period 1 and significant at the 0.001 level for all other time periods. Mean fluorescence levels were consistently higher for samples exposed to UF particles.

It can be seen in Fig. 6 that fluorescence values increase over time in both conditions. However, the increase appears to be both more gradual and more linear in samples exposed to C particles than in samples exposed to UF particles. This data corroborates what was seen in the TEM images since the PL formation should correspond to the mROS generation.

### Expression of inflammatory cytokines

As a way of elucidating the differences in expression of inflammatory cytokines based on exposure to different sized CS particles, we exposed AM to UF, S, R, and C particles for two-, four- and eight-hour exposure periods. A Luminex 200 (Bio-Plex200, Bio-Rad) was used to measure the following cytokines in cell culture supernatant: FGF-basic (fibroblast growth factor 2); GM-CSF (granulocyte-macrophage colony-stimulating factor or colony stimulating factor 2); IFN-γ (interferon-gamma); IL-1α, IL-1β, KC (melanoma growth stimulating activity, alpha); IL2, IL4, IL5, IL6, IL10, IL12B, IL13, IL17A, IP-10 (CXCL10); MCP-1 (monocyte chemoattractant protein 1); MIP-1 alpha (macrophage inflammatory protein 1-alpha); MIG (CXCL9); TNF-α (tumor necrosis factor alpha); and VEGFA (vascular endothelial growth factor A). The data show that for each exposure period the positive control resulted in significantly elevated expression of the measured cytokines when compared to the CS particle exposures and the negative controls.

**Fig. 7:**
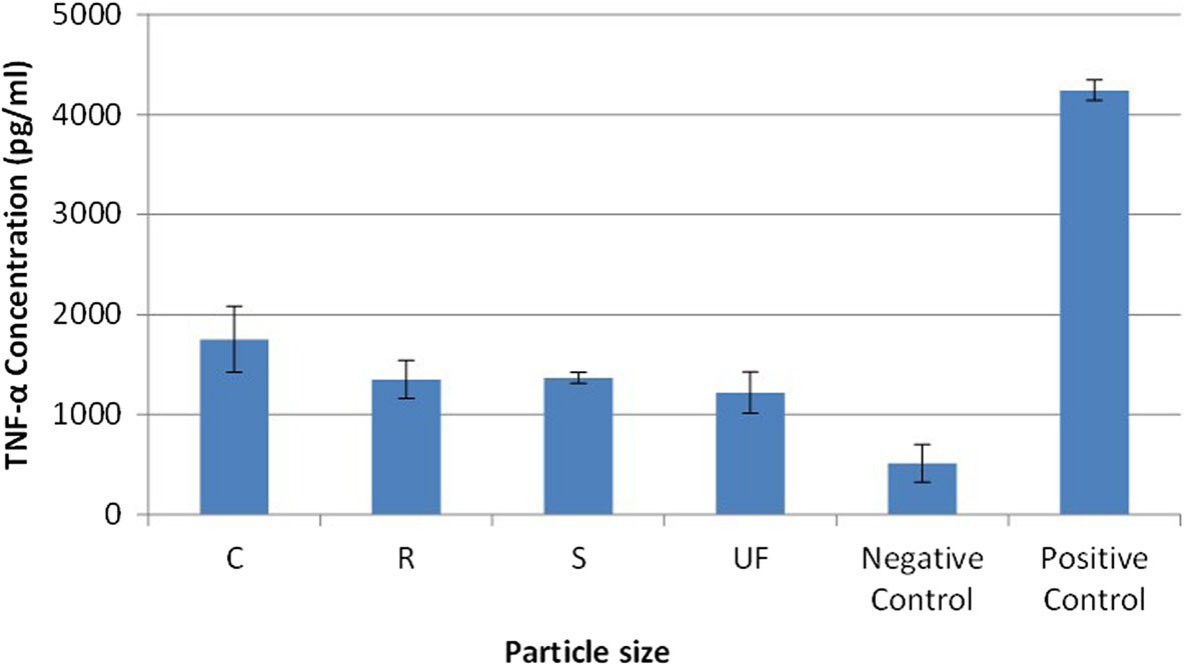
TNF-α expression after 2-h exposure to four sizes of Crystalline Silica, showing that for a 2-h exposure period each particle size creates a significant increase in expression of TNF-α when compared to the negative control; however, there was no significant difference on the expression of TNF-α based on particle size

The results from the Bio-Plex analysis for the two-hour exposure period are shown for TNF-α in Fig. 7. For this exposure period each CS particle size showed a significant increase in expression of TNF-α when compared to the negative control; however, there was no significant difference on the expression of TNF-α based on CS particle size. Other cytokines measured in the media from the CS particle exposures included IL-5, IL-10, IP-10, MCP-1, and MIP-1A, but no statistical difference based on CS particle size was found for any of these inflammatory cytokines (Data not shown).

**Fig. 8:**
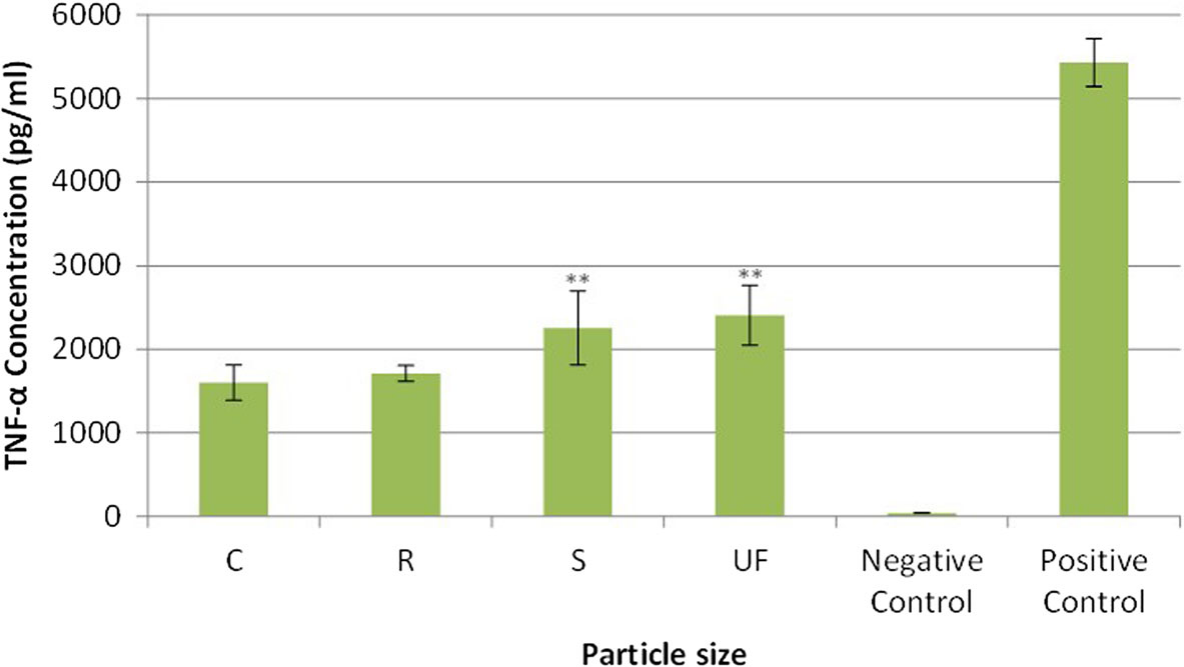
TNF-α expression after 4-h exposure to four sizes of Crystalline Silica, showing that for a 4-h exposure period each particle size created a significant increase (*p*-value < = 0.005) in expression of TNF-α when compared to the negative control samples. In addition, both the UF and the S particles showed a statistically significant increase in expression of TNF-α when compared to both the C and R particles (p-value = 0.005). ** Significant difference from C exposure at *p*-value = 0.005

The results from the Bio-Plex analysis for the four-hour exposure period are presented for TNF-α in Fig. 8. At this exposure period each CS particle size showed a significant increase (*p*-value < = 0.005) on expression of TNF-α when compared to the negative control samples. In addition, both the UF and the S particles showed a statistically significant increase in expression of TNF-α when compared to both the C and R particles. Other cytokines measured during this analysis showing a significant difference of expression from exposure to UF versus C particles are as follows: MCP-1 (*p*-value = 0.07); IL-12 (*p*-value = 0.003); IL-5 (*p*-value = 0.002; and IL-6 (*p*-value = 0.013). These data also corroborate those seen in Figs. 2 through 4, since the cytokine production would be expected to be delayed when compared to both PL formation and mROS generation.

**Fig. 9:**
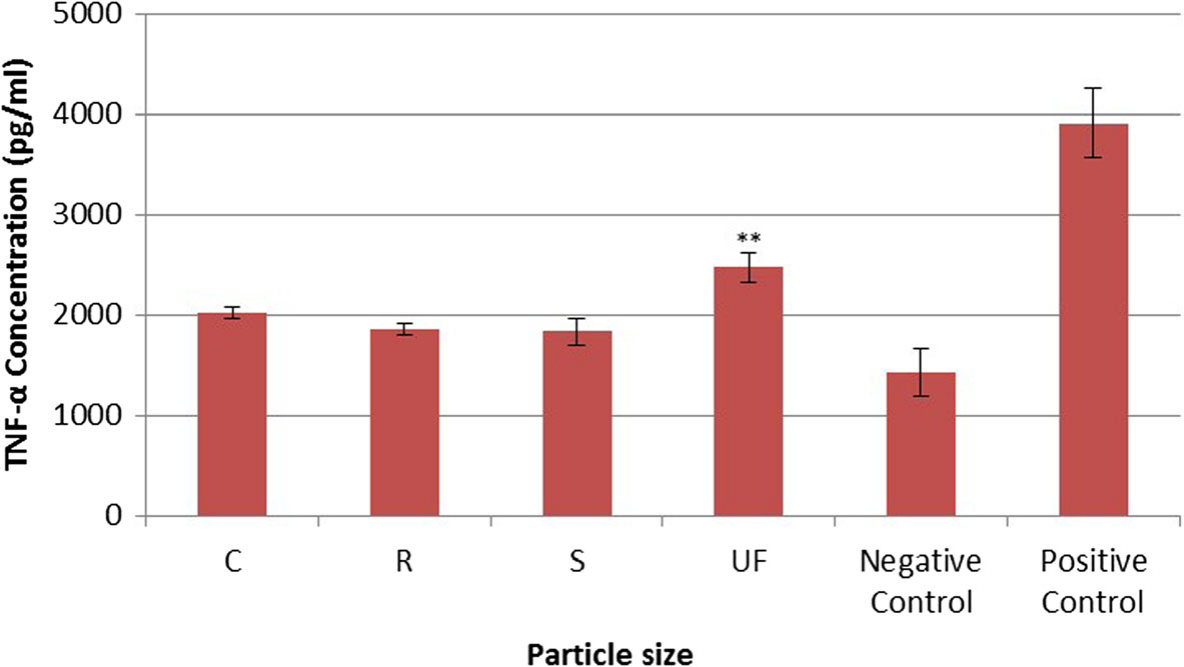
TNF-α expression after 8-h exposure to four sizes of Crystalline Silica, showing that for an 8-h exposure period, each particle size created a significant increase (*p*-value < = 0.005) in the expression of TNF-α when compared to the negative control samples. In addition, the UF particles were found to significantly enhance the expression of TNF-α when compared to the other three particle sizes (*p*-value = 0.0001). ** Significant difference from C exposure at *p*-value = 0.0001

The results from the Bio-Plex analysis for the eight-hour exposure period are presented for TNF-α in Fig. 9. At this exposure period, each CS particle size showed a significant increase (*p*-value < = 0.005) on the expression of TNF-α when compared to the negative control samples. Also, the UF particles were found to significantly enhance the expression of TNF-α when compared to the other three particle sizes.

## Discussion

This study aimed to understand the effect of different sized crystalline silica particles on the activation and response of murine alveolar macrophages. For this study, the CS was divided into four distinct size ranges-C (4 μm), R (2 μm), S (0.7 μm), and UF (0.3 μm)-using a multi-cyclone sampling array. This method is capable of separating airborne occupational aerosols into distinct size ranges as described in the Methods section. The CS particles at each size range were well defined both in the air and in the culture media.

The results of this study show a consistent relationship between CS particle sizes and AM activation, as measured by TEM imaging, mROS generation, and inflammatory cytokine expression. The TEM data show that a greater number of UF particles are phagocytized in a shorter time period than C particles. In addition, the number of PLs resulting from the UF exposure, the size of the PLs, and the number of particles in each of the PLs is increased for the UF particles when compared to the C particles. The mROS data show both a faster and more intense response from exposure to UF particles versus C particles. Finally, the cytokine measurements showed that UF particles resulted in a significant increase in TNF-α expression from the AM at four- and eight-hour exposures when compared to C exposure. TNF-α expression is a primary indicator of AM activation [7, 32]. Other cytokines measured during this analysis also show a significant difference in expression between UF exposure versus C exposure at four hours, including MCP-1, IL-12, IL-5, and IL-6. These results agree with previous work discussing cytokine release and AM activation due to silica exposure [28, 33, 34].

Much work, both in vivo and in vitro, is evident in the literature comparing the effect of particle size on biological outcomes. This previous work usually agrees with our data that smaller particles create an enhanced biological response. Typically, this previous work used engineered particles produced in laboratories at specific sizes, such as amorphous silica, titanium dioxide (TiO_2_), or gold [18, 29, 35, 36]. Oberdorster et al. observed significantly greater pulmonary inflammatory response to ultrafine (20-nm) TiO_2_ in rats and mice when compared to larger particles (250-nm) [37]. Sager et al. found that ultrafine (21-nm) TiO_2_ particles caused significantly greater inflammation and were more cytotoxic than fine (1-μm) TiO_2_ particles when instilled into rats [38]. Also, Leclerc et al. investigated in vitro the rate of macrophage uptake and toxicity of fluorescent silica particles ranging from 850 to 150 nm and found that the smallest particles were internalized in greater quantities [18].

Despite the above work, there are relatively few studies comparing biological effects from different sizes of crystalline silica. Wiessner et al. showed that a 1-μm fraction was more lytic to red blood cell membranes than larger fractions; however, the larger size fractions resulted in a greater in vivo inflammatory response [13]. In this study, the 1-μm fraction was the smallest used. Kajiwara et al. demonstrated that a 1.8-μm size fraction more intensely affected the lungs of mice than a 0.7-μm size fraction [16]. The major differences between our current study and these previous studies are the size ranges of the particles used for exposure and the procedure for separating the particles into these distinct size ranges. The particles sizes used in these previous studies ranged from 1.8 to 0.7 μm whereas the range for our study was 4 μm to 0.3 μm. Wang et al. showed that silica particles in the size fraction <200 nm were both cytotoxic and genotoxic to human cells [17]. However, this study did not compare the effects of particle size and did not use AM, so no activation criteria were reported.

Each of these previous studies used separation technologies that allowed particle crossover between size ranges such that smaller particles are present in larger particle ranges and vice versa. The biological effects measured in these experiments may have been influenced by the presence of these crossover particles. In the present study, the MCSA was used to separate the crystalline silica particles into distinct size ranges with little crossover between ranges. In addition, the MCSA method allows for the particle separation to occur from the air in concentrations found in occupational environments. Because the particles are separated from the air, they are unaffected by the handling steps necessary for other separation techniques [23].

In addition to showing the significance of particle size on the activation of AMs, our study also sheds some light on the molecular mechanisms behind this activation. The inflammatory effect was shown to be mediated from the recruitment of the NALP3 inflammosome [30, 39, 40]; however, the mechanism of this recruitment and silica-induced toxicity is still in question [27, 35]. In this study we provide data to indicate that mROS production occurs very early in the AM reaction to silica and coincides with the PL formation and swelling in the AM. In addition, the cytokine expression is delayed when compared to the mROS production. In our data, we see a significant difference in mROS generation based on exposure to UF particles versus C particles, with the greatest difference between 2 and 2.5 h. However, at the two-hour time point we see no difference in cytokine expression between these particle sizes. By four hours, a significant difference in cytokine expression between the UF and C particle exposures appears. The TEM images show an increased number of UF particles being phagocytized at two hours and some swelling of the PLs is occurring. By the four-hour time period, there are noticeably more PLs and they are noticeably swelling, when compared to the same time after C particle exposure and as the number of PLs increases the generation of mROS increases. Cassel et al. showed that cellular ROS signaling is occurring upstream of the NALP3 inflammasome activation. Dostert et al. suggested that these inflammasome activating cellular ROS are generated by NADPH oxidase complexes on the phagolysosome membrane [40].

The above information, along with the data presented in this study, lends further definition to the pathway recently outlined by Lueng [4]. In this process, cellular ROS is generated by both the mitochondria and the NAPDH oxidase after phagocytosis and this process happens quickly after exposure. The cellular ROS then activates the NALP3 inflammasome, leading to expression of inflammatory cytokines. Our data supports this mechanism since the UF particles are more quickly incorporated into PLs and there are a greater number of PLs formed, corresponding to an increase in mROS generation and ultimately to elevated expression of inflammatory cytokines. Since only mROS was measured in this study, our data suggest that the increase in cellular ROS, as described earlier, is at least partly caused by generation of ROS in the mitochondria.

A second possibility resulting in inflammation is the activation of the NFκB pathway by cellular ROS. This pathway has been shown to be activated by CS [14, 41], resulting in the expression of TNF-α. Our data also supports this pathway, since TNF-α production continues to increase as the number of PLs and mROS generation increase in the AM. Scarfi et al. add a slightly different perspective on this mechanism [32] by showing that cellular ROS generation and TNF-α expression occur in the absence of phagocytosis. In this case the plasma membrane appears to play a key role in the cellular ROS generation.

Taken together, these data suggest that two potential pathways, independent of each other, lead to the inflammatory response in AM. The first pathway was suggested by Scarfi et al. where the cellular ROS generation results from the CS interaction with the plasma membrane prior to phagocytosis, as a result of lipid peroxidation. Our data show mROS generation occurring with the introduction of the CS particles and TNF-α expression measured at two hours after exposure. In addition, our data show that the mROS production and TNF-α expression increase more quickly corresponding to an increase in the number of PLs formed. In this case, the mROS generation would result from the PL membrane disruption caused by CS and add to the cellular ROS generated from the plasma membrane. Since there are a greater number of PLs created in response to the UF particle exposure, the difference in mROS generation between the two exposure scenarios, as well as the resulting TNF-α expression, should increase as the PLs are formed. This is exactly what our data show. In this slightly delayed scenario the inflammatory response would result from PL generated mROS either through the activation of the NALP3 inflammasome or through the initiation of the NFκB cascade. In both scenarios, the UF particles should cause an enhanced response due first from plasma membrane interaction with a larger number of CS particles and then from the formation of a greater number of PLs after phagocytosis.

## Conclusions

The aim of this research was to determine the effect of the size of crystalline silica particles on the activation of macrophages. This study provided novel data showing that UF silica particles enhance the activation of AM when compared to larger silica particles usually represented in in vitro and in vivo research. These data identified differences in particle uptake and formation of subcellular organelles based on particle size. In addition, these data show that the smallest particles, with a geometric mean of 0.3 μm, significantly increase the generation of mitochondrial ROS and the expression of cytokines when compared to larger crystalline silica particles, with a geometric mean of 4.1 μm. However, these data are only meaningful if it can be shown that UF silica particles are a relevant occupational exposure. Recently it was shown that nanoparticles, ranging from 850 to 100 nm inhaled by rats were found in the alveolar macrophages of these animals [42] providing evidence that particles as small as 100 nm can be incorporated into AMs. Furthermore, it has been shown that in occupational environments there is significant variation in particle size and size-related silica content [8], and that as the average particle size of the sample decreases, the percent silica of the sample may increase [43]. Because such substantial differences in particle size and CS content of occupational aerosols have been shown to occur and because we have shown in this study that UF CS particles enhance the activation of AM compared to larger CS particles, more research is needed to more fully define this exposure and potential adverse biological outcomes of UF silica particles. In addition, the results of this study lend support to the growing chorus of researchers calling for regulations based on metrics capable of measuring UF and nanoparticles, to help better protect workers from this exposure.

## Abbreviations

AM: Alveolar macrophage
ATCC: American type tissue culture collection
C: Coarse
CS: Crystalline silica
DMEM: Dulbecco’s modified eagle’s medium
FBS: Fetal bovine serum
MCSA: Multi-cyclone sampling array
mROS: Mitochondrial reactive oxygen species
OSHA: United states occupational safety and health administration
PBS: Phosphate buffered saline
PL: Phagolysosome
R: Respirable
RCF: Relative centrifugal force
ROS: Reactive oxygen species
S: Submicron
SEM: Scanning electron microscope
TEM: Transmission electron microscopy
UF: Ultrafine particles

## References

[1] NIOSH (2002). NIOSH Hazard Review; Health Effects of Occupational Exposure to Respirable Crystalline Silica.

[2] Greenberg MI, Waksman J, Curtis J (2007). Silicosis: a review. Dis Mon.

[3] The Global Occupational Health Network Newsletter: elimination of silicosis [http://www.who.int/occupational_health/publications/newsletter/gohnet12e.pdf]. Accessed 9 Dec 2016.

[4] Leung CC, Yu IT, Chen W (2012). Silicosis. Lancet.

[5] Work-Related Lung Disease Surveillance System (eWoRLD); Silicosis Mortality [http://www2a.cdc.gov/drds/worldreportdata/SubsectionDetails.asp?ArchiveID=1&SubsectionTitleID=8]. Accessed 9 Dec 2016.

[6] Silicosis Fact Sheet N° 238 [http://www.nzdl.org/gsdlmod?e=d-00000-00---off-0cdl--00-0----0-10-0---0---0direct-10---4-------0-1l--11-en-50---20-about---00-0-1-00-0--4----0-0-11-10-0utfZz-8-00&cl=CL1.242&d=HASHf58c7c472d6ca58330314f.2&x=1]

[7] Huaux F (2007). New developments in the understanding of immunology in silicosis. Curr Opin Allergy Clin Immunol.

[8] Sirianni G, Hosgood HD, Slade MD, Borak J (2008). Particle size distribution and particle size-related crystalline silica content in granite quarry dust. J Occup Environ Hyg.

[9] Beaudry C, Lavoue J, Sauve JF, Begin D, Senhaji Rhazi M, Perrault G, Dion C, Gerin M (2013). Occupational exposure to silica in construction workers: a literature-based exposure database. J Occup Environ Hyg.

[10] Hall RM, Achutan C, Sollberger R, McCleery RE, Rodriguez M (2013). Exposure assessment for roofers exposed to silica during installation of roof tiles. J Occup Environ Hyg.

[11] McKinney W, Chen B, Schwegler-Berry D, Frazer DG (2013). Computer-automated silica aerosol generator and animal inhalation exposure system. Inhal Toxicol.

[12] Sauve JF, Beaudry C, Begin D, Dion C, Gerin M, Lavoue J (2013). Silica exposure during construction activities: statistical modeling of task-based measurements from the literature. Ann Occup Hyg.

[13] Wiessner JH, Mandel NS, Sohnle PG, Mandel GS (1989). Effect of particle size on quartz-induced hemolysis and on lung inflammation and fibrosis. Exp Lung Res.

[14] Fubini B, Hubbard A (2003). Reactive oxygen species (ROS) and reactive nitrogen species (RNS) generation by silica in inflammation and fibrosis. Free Radic Biol Med.

[15] Bodo M, Muzi G, Bellucci C, Lilli C, Calvitti M, Lumare A, Dell’Omo M, Gambelunghe A, Baroni T, Murgia N (2007). Comparative in vitro studies on the fibrogenic effects of two samples of silica on epithelial bronchial cells. J Biol Regul Homeost Agents.

[16] Kajiwara T, Ogami A, Yamato H, Oyabu T, Morimoto Y, Tanaka I (2007). Effect of particle size of intratracheally instilled crystalline silica on pulmonary inflammation. J Occup Health.

[17] Wang JJ, Sanderson BJ, Wang H (2007). Cytotoxicity and genotoxicity of ultrafine crystalline SiO2 particulate in cultured human lymphoblastoid cells. Environ Mol Mutagen.

[18] Leclerc L, Rima W, Boudard D, Pourchez J, Forest V, Bin V, Mowat P, Perriat P, Tillement O, Grosseau P (2012). Size of submicrometric and nanometric particles affect cellular uptake and biological activity of macrophages in vitro. Inhal Toxicol.

[19] Churg A, Brauer M (2000). Ambient atmospheric particles in the airways of human lungs. Ultrastruct Pathol.

[20] Donaldson K, MacNee W (2001). Potential mechanisms of adverse pulmonary and cardiovascular effects of particulate air pollution (PM10). Int J Hyg Environ Health.

[21] Knol AB, de Hartog JJ, Boogaard H, Slottje P, van der Sluijs JP, Lebret E, Cassee FR, Wardekker JA, Ayres JG, Borm PJ (2009). Expert elicitation on ultrafine particles: likelihood of health effects and causal pathways. Part Fibre Toxicol.

[22] Pope CA, Burnett RT, Krewski D, Jerrett M, Shi Y, Calle EE, Thun MJ (2009). Cardiovascular mortality and exposure to airborne fine particulate matter and cigarette smoke: shape of the exposure-response relationship. Circulation.

[23] Mischler SE, Cauda EG, Di Giuseppe M, Ortiz LA (2013). A multi-cyclone sampling array for the collection of size-segregated occupational aerosols. J Occup Ind Hyg.

[24] Monteiller C, Tran L, MacNee W, Faux S, Jones A, Miller B, Donaldson K (2007). The pro-inflammatory effects of low-toxicity low-solubility particles, nanoparticles and fine particles, on epithelial cells in vitro: the role of surface area. Occup Environ Med.

[25] Mossman BT, Churg A (1998). Mechanisms in the pathogenesis of asbestosis and silicosis. Am J Respir Crit Care Med.

[26] Rimal B, Greenberg AK, Rom WN (2005). Basic pathogenetic mechanisms in silicosis: current understanding. Curr Opin Pulm Med.

[27] Hamilton RF, Thakur SA, Holian A (2008). Silica binding and toxicity in alveolar macrophages. Free Radic Biol Med.

[28] Gozal E, Ortiz LA, Zou X, Burow ME, Lasky JA, Friedman M (2002). Silica-induced apoptosis in murine macrophage: involvement of tumor necrosis factor-alpha and nuclear factor-kappaB activation. Am J Respir Cell Mol Biol.

[29] Sandberg WJ, Lag M, Holme JA, Friede B, Gualtieri M, Kruszewski M, Schwarze PE, Skuland T, Refsnes M (2012). Comparison of non-crystalline silica nanoparticles in IL-1beta release from macrophages. Part Fibre Toxicol.

[30] Cassel SL, Eisenbarth SC, Iyer SS, Sadler JJ, Colegio OR, Tephly LA, Carter AB, Rothman PB, Flavell RA, Sutterwala FS (2008). The Nalp3 inflammasome is essential for the development of silicosis. Proc Natl Acad Sci U S A.

[31] Rosner B (1990). Fundamentals of Biostatistics.

[32] Scarfi S, Magnone M, Ferraris C, Pozzolini M, Benvenuto F, Benatti U, Giovine M (2009). Ascorbic acid pre-treated quartz stimulates TNF-alpha release in RAW 264.7 murine macrophages through ROS production and membrane lipid peroxidation. Respir Res.

[33] Driscoll KE, Hassenbein DG, Carter JM, Kunkel SL, Quinlan TR, Mossman BT (1995). TNF alpha and increased chemokine expression in rat lung after particle exposure. Toxicol Lett.

[34] Balduzzi M, Diociaiuti M, De Berardis B, Paradisi S, Paoletti L (2004). In vitro effects on macrophages induced by noncytotoxic doses of silica particles possibly relevant to ambient exposure. Environ Res.

[35] Winter M, Beer HD, Hornung V, Kramer U, Schins RP, Forster I (2011). Activation of the inflammasome by amorphous silica and TiO2 nanoparticles in murine dendritic cells. Nanotoxicology.

[36] Downs TR, Crosby ME, Hu T, Kumar S, Sullivan A, Sarlo K, Reeder B, Lynch M, Wagner M, Mills T, Pfuhler S (2012). Silica nanoparticles administered at the maximum tolerated dose induce genotoxic effects through an inflammatory reaction while gold nanoparticles do not. Mutat Res.

[37] Oberdorster G, Finkelstein JN, Johnston C, Gelein R, Cox C, Baggs R, Elder AC (2000). Acute pulmonary effects of ultrafine particles in rats and mice. Res Rep Health Eff Inst.

[38] Sager TM, Kommineni C, Castranova V (2008). Pulmonary response to intratracheal instillation of ultrafine versus fine titanium dioxide: role of particle surface area. Part Fibre Toxicol.

[39] Hornung V, Bauernfeind F, Halle A, Samstad EO, Kono H, Rock KL, Fitzgerald KA, Latz E (2008). Silica crystals and aluminum salts activate the NALP3 inflammasome through phagosomal destabilization. Nat Immunol.

[40] Dostert C, Petrilli V, Van Bruggen R, Steele C, Mossman BT, Tschopp J (2008). Innate immune activation through Nalp3 inflammasome sensing of asbestos and silica. Science.

[41] Cox LA (2011). An exposure-response threshold for lung diseases and lung cancer caused by crystalline silica. Risk Anal.

[42] Morfeld P, Treumann S, Ma-Hock L, Bruch J, Landsiedel R (2012). Deposition behavior of inhaled nanostructured TiO2 in rats: fractions of particle diameter below 100 nm (nanoscale) and the slicing bias of transmission electron microscopy. Inhal Toxicol.

[43] Page SJ (2003). Comparison of coal mine dust size distributions and calibration standards for crystalline silica analysis. AIHA J (Fairfax, Va).

